# Relationship between systemic immune-inflammation index and all-cause mortality in stages IIIB–IV epidermal growth factor receptor-mutated lung adenocarcinoma

**DOI:** 10.3389/fendo.2025.1698317

**Published:** 2025-11-05

**Authors:** Chi Zhang, Chao Yang

**Affiliations:** ^1^ Department of Oncology, Anhui Chest Hospital, Hefei Anhui, China; ^2^ Anhui Medical University Clinical College of Chest, Hefei Anhui, China; ^3^ Department of Urology, Anhui Provincal Children’s Hospital, Hefei Anhui, China

**Keywords:** lung adenocarcinoma, systemic immune-inflammation index, epidermal growth factor receptor mutation, prognosis, all-cause mortality

## Abstract

**Background:**

This study investigates the relationship between the systemic immune-inflammation index (SII) and all-cause mortality (ACM) risk in individuals with stages IIIB–IV epidermal growth factor receptor (EGFR)-mutated lung adenocarcinoma.

**Methods:**

The clinical data of 187 individuals with stages IIIB–IV EGFR-mutated lung adenocarcinoma from Anhui Chest Hospital, collected from June 2017 to December 2023, were retrospectively analyzed. SII was calculated as platelet count × neutrophil count/lymphocyte count. The receiver operating characteristic (ROC) curve was employed to determine the optimal threshold SII, and individuals were classified as low and high SII groups. ACM serves as the primary endpoint. Univariate and multivariate analyses were conducted using Cox proportional hazards models. The robustness of the findings was tested by subgroup and sensitivity analyses.

**Results:**

The ACM risk was notably elevated in the high SII group (*p* = 0.001) compared with the low SII group. Multivariate Cox analysis demonstrated that SII can independently predict poor prognosis. In the fully adjusted model, compared with the low SII group, the ACM risk was 1.985 times higher in the high SII group (hazard ratio [HR] = 1.985; 95% confidence interval [CI] = 1.216–3.240; *p* = 0.006). Subgroup analyses showed that SII was more strongly associated with ACM risk in men (HR = 3.245; *p* = 0.005), and this relationship was also significant among female patients (HR = 2.036; *p* = 0.048). In individuals aged ≥ 65 years, a high SII was significantly associated with an elevated ACM risk (HR = 2.675; *p* = 0.004). No such relationship was observed in individuals aged under 65. Sensitivity analyses indicated that high SII remained significantly correlated with elevated ACM risk after excluding individuals with special types of adenocarcinoma, stage III lung adenocarcinoma, or diabetes (all *p<* 0.05), supporting its potential as an independent prognostic indicator. ROC curve analysis demonstrated that SII had a moderate predictive ability for ACM, with an AUC of 0.669 (95% CI = 0.527–0.812; *p* = 0.021).

**Conclusion:**

Elevated SII is an independent biomarker for predicting ACM in individuals with stages IIIB–IV EGFR-mutated lung adenocarcinoma, with a stronger predictive value in male and older populations.

## 1 Introduction

Lung adenocarcinoma is a prevalent malignancy worldwide (1). Due to its high mortality rate and complex biological characteristics, the treatment and prognosis assessment of this disease remain a major focus of clinical research. With rapid advances in immunotherapies and precision medicine, the role of epidermal growth factor receptor (EGFR) mutations in lung adenocarcinoma has become increasingly recognized. Consequently, EGFR has emerged as a key therapeutic target (2). However, substantial differences exist in treatment responses and survival outcomes among patients with stages IIIB–IV EGFR-mutated lung adenocarcinoma. Consequently, more reliable biomarkers are needed to guide treatment decisions and prognostic assessments in clinical practice. Previous studies (3) have shown that inflammation can significantly influence tumor angiogenesis, metastasis, and overall cancer progression. The systemic immune-inflammation index (SII) has emerged as a valuable biomarker for predicting outcomes in various cancer types (4). This indicator can assess the body’s immune-inflammatory status by integrating platelet, neutrophil, and lymphocyte counts (5). Existing research has demonstrated that SII is both sensitive and specific for assessing lung cancer (LC) prognosis. It provides a comprehensive measure of the balance between inflammatory cytokines and immune status. Moreover, it can effectively predict recurrence, metastasis, and overall prognosis in various malignancies, including pancreatic, breast, and bladder cancers (6).

By analyzing the relationship between SII and ACM in patients with stages IIIB–IV EGFR-mutated lung adenocarcinoma, this study aims to provide a foundation for developing a prognostic tool to guide individualized treatment. Evaluating the interplay between SII, patient clinical characteristics, treatment responses, and survival outcomes may offer valuable guidance for clinical management and further support the use of SII as a potential biomarker for assessing prognosis in this population.

## 2 Materials and methods

### 2.1 Study population

This study consecutively enrolled patients with stages IIIB–IV EGFR-mutated lung adenocarcinoma who received systemic anticancer therapy at Anhui Chest Hospital between June 2017 and December 2023, aiming to minimize potential selection bias. Cases were identified from the institutional cancer registry and electronic medical records by two independent investigators. Records that were missing or incomplete were excluded from the analysis.

The clinical data of 187 individuals with stages IIIB–IV EGFR-mutated lung adenocarcinoma from Anhui Chest Hospital between 28 June 2017 and 18 December 2023 were retrospectively collected. Inclusion criteria were as follows: (i) lung adenocarcinoma diagnosed cytologically or histologically; (ii) tumor–node–metastasis (TNM) stages IIIB–IV; (iii) EGFR gene mutation. Exclusion criteria included: (i) presence of other cancers; (ii) severe infection, autoimmune diseases, or other diseases affecting inflammatory markers; (iii) use of medications that might influence SII (such as corticosteroids and non-steroidal anti-inflammatory drugs); (iv) missing SII data prior to chemotherapy or targeted therapy; (v) incomplete follow-up data. Approval from the Ethics Committee of Anhui Chest Hospital was obtained. Given that the data used were anonymized, informed consent was waived.

Patients with histologically or cytologically confirmed lung adenocarcinoma harboring EGFR mutations (stages IIIB–IV) were consecutively enrolled. EGFR mutation status was determined by polymerase chain reaction/amplification refractory mutation system (PCR/ARMS) or next-generation sequencing (NGS) performed in our hospital or reported from external certified laboratories. Any primary EGFR mutation identified (including common sensitizing mutations such as exon 19 deletion and L858R, as well as rare or complex variants) was categorized as EGFR-mutated. However, detailed mutation subtype data were incomplete for some patients; therefore, subgroup analyses by mutation subtype (e.g., exon 19 deletion, L858R, T790M, or others) were not conducted.

### 2.2 Data collection and definitions

Clinical data of eligible individuals were derived from their admission records and electronic medical records. Collected data included disease-related information, demographic characteristics, lifestyle factors, medical history, physical measurements, and laboratory test results. Demographics included gender (woman or man) and age (at the time of diagnosis). Disease-related information were pathological type (adenocarcinoma [ADC] or special types of ADC), clinical stage (stage III or IV), metastatic sites (intrapulmonary metastasis only or extrapulmonary metastasis included), treatment type (targeted therapy alone or combination therapy), treatment response (partial response or stable or progressive disease based on response evaluation criteria in solid tumors, version 1.1 [RECIST 1.1]), and disease progression (categorized as progressed or not according to follow-up data).

In terms of lifestyle factors, smoking history was defined as a history of sustained smoking, regardless of current smoking status. A family history was determined as the presence of LC or other cancers in first-degree relatives. Comorbid conditions, including hypertension (HP) (according to previous diagnosis or current use of antihypertensive medications), chronic obstructive pulmonary disease (COPD) (according to established diagnosis or pulmonary function tests), diabetes mellitus (DM) (according to clinical diagnosis or current use of antidiabetic medications), and tuberculosis (based on prior diagnosis and antituberculosis treatment), were recorded as present or absent.

Body measurements included weight and height. The body mass index (BMI) was calculated as weight/height². Moreover, diastolic blood pressure (DBP) and systolic blood pressure (SBP) were documented in millimeters of mercury (mmHg) at admission.

Laboratory test parameters encompassed (i) blood cell counts, including neutrophils, white blood cells (WBC), lymphocytes, platelets, and monocytes; (ii) metabolic indicators, including fasting blood glucose (FBG), total cholesterol (TC), uric acid (UA), high-density lipoprotein cholesterol (HDL-C), triglycerides (TG), and low-density lipoprotein cholesterol (LDL-C); (iii) renal function indicators, such as blood urea nitrogen (BUN) and serum creatinine (Cr); (iv) electrolytes, such as serum calcium (SC), serum sodium (SS), and serum potassium (SP); (v) hepatic function indicators, encompassing aspartate aminotransferase (AST), total bilirubin (TBil), and alanine aminotransferase (ALT).

Two researchers separately extracted and cross-checked the data to ensure accuracy and consistency. Categorical variables were defined and classified according to standardized criteria to ensure the scientific validity and reproducibility of findings.

### 2.3 SII definition

The SII, an emerging immune-inflammatory marker, reflects the intensity of both inflammatory status and immune response. It was calculated as platelet count × neutrophil count/lymphocyte count (unit: × 10^9^/L). The first blood test result before systemic anti-cancer treatment was used to calculate the SII value. Given the right-skewed distribution of the original SII data, log transformation (Log_10_SII) was applied in the Cox proportional hazards (CPH) models to improve normality and enhance model stability.

In this study, SII was analyzed both as a categorical variable—by dividing individuals into low- and high-SII groups based on the optimal cutoff value (411.29)—for survival comparison, and as a continuous variable (SII or log10SII) in the Cox proportional hazards (CPH) models to comprehensively explore its association with all-cause mortality (ACM).

Categorization based on a cutoff value enhances clinical interpretability and facilitates risk stratification. The optimal cutoff value of SII (411.29) was determined using receiver operating characteristic (ROC) curve analysis combined with Youden’s index, which identifies the point achieving the best balance between sensitivity and specificity. This objective and data-driven method reflects the most effective threshold for distinguishing survival risk within our cohort.

Moreover, the use of ROC-based cutoff determination for SII is consistent with previous studies. For instance, Deng et al. (7) and Tong et al. (8) applied similar ROC approaches in patients with non-small cell lung cancer (NSCLC) and reported cutoff values ranging from approximately 400 to 660. The cutoff value identified in our study falls within this range, demonstrating methodological consistency and external comparability.

### 2.4 Follow-up

This study adopted a retrospective cohort design with long-term follow-up through multiple channels, including electronic medical records, discharge summaries, outpatient follow-ups, and telephone interviews. Follow-up began on the date the patient first received systemic anticancer treatment (such as targeted therapy or combination therapy), and ended on 1 January 2025, or at the time of death. The primary endpoint was ACM. All death events were verified by two researchers based on hospital records, death certificates, or reports from family members to maintain the accuracy of the endpoint.

### 2.5 Statistical analysis

SPSS 26.0 software was employed to carry out data analysis. Baseline characteristics were first summarized using descriptive statistics. Continuous variables that were normally distributed were represented by mean ± standard deviation $\left(\bar{x}\pm s\right)$ and compared between groups using independent-samples *t*-tests. Nonnormally distributed continuous variables were presented as median (interquartile range) and compared using the Mann–Whitney *U* test. Categorical variables were reported as frequencies (percentages) and analyzed using either the *χ*² test or Fisher’s exact test, as appropriate.

The ROC curve was performed to determine the optimal cutoff value of the SII, based on which patients were classified into low and high SII groups. Kaplan–Meier (KM) survival curves for ACM were constructed for both groups, and the log-rank test was used to compare survival distributions. Univariate and multivariate CPH models were employed to examine the association between SII and ACM. Potential confounding factors, including demographics, comorbidities, and laboratory markers, were progressively included in three adjusted models. Hazard ratios (HRs) and their 95% confidence intervals (CIs) were calculated to quantify associations.

Subgroup analyses stratified by age and sex, along with sensitivity analyses excluding specific patient groups, were carried out to examine the robustness of the results. A two-sided *p*-value below 0.05 was considered statistically significant. ROC curve analysis was conducted to evaluate the discriminative ability of the SII for predicting ACM.

To ensure precision, HRs and 95% confidence intervals were reported to three decimal places. The multivariate Cox proportional hazards model was adjusted for variables significant in univariate analyses (*p* < 0.1), including diabetes, pathological type, fasting glucose, WBC, and serum calcium.

## 3 Results

### 3.1 Baseline characteristics according to SII cutoff value

As illustrated in **Table 1**, compared with the low SII group, a notably greater ACM risk (*p* = 0.001), elevated levels of platelet count (*p* < 0.001), neutrophil count (*p* < 0.001), WBC (*p* < 0.001), monocyte count (*p* = 0.009), and lower level of lymphocyte count (*p* = 0.009) was found in the high SII group. However, there was no substantial differences between the two groups regarding clinical stage, gender, metastasis site, treatment modality, pathological type, family history, age, treatment response, disease progression, smoking status, HP, DM, COPD, pulmonary tuberculosis (PTB), BMI, SBP, DBP, hemoglobin, BUN, albumin, ALT, HDL-C, AST, TBil, UA, TC, TG, Cr, LDL-C, FBG, SP, SS, and SC (*p* > 0.05).

**Table 1:** Baseline characteristics stratified by the optimal cutoff value of SII.

Variable	Total population	Low SII group	High SII group	P-value
Age, years	65.00 (55.00, 72.00)	69.00 (58.00, 73.00)	64.00 (54.00, 72.00)	0.209
Gender, n (%)				0.227
Male	92 (49.2)	14 (40.0)	78 (51.3)	
Female	95 (50.8)	21 (60.0)	74 (48.7)	
Family history, n (%)				0.567
Positive	183 (97.9)	34 (97.1)	149 (98.0)	
Negative	4 (2.1)	1 (2.9)	3 (2.0)	
Pathological type, n (%)				0.421
Adenocarcinoma	161 (86.1)	32 (91.4)	129 (84.9)	
Special types of adenocarcinoma	26 (13.9)	3 (8.6)	23 (15.1)	
Clinical stage, n (%)				0.100
Stage III	15 (8)	3 (8.6)	12 (7.9)	
Stage IV	172 (92)	32 (91.4)	140 (92.1)	
Site of metastasis, n (%)				0.375
Intrapulmonary metastasis only	105 (56.1)	22 (62.9)	83 (54.6)	
Extrapulmonary metastasis included	82 (43.9)	13 (37.1)	69 (45.4)	
Treatment modality, n (%)				0.751
Targeted therapy alone	106 (56.7)	19 (54.3)	87 (57.2)	
Combination therapy	81 (43.3)	16 (45.7)	65 (42.8)	
Treatment response, n (%)				0.078
PR	172 (92.0)	35 (100.0)	137 (90.1)	
SD+PD	15 (8.0)	0 (0.0)	15 (9.9)	
Disease progression, n (%)				0.152
No	23 (12.3)	7 (20.0)	16 (10.5)	
Yes	164 (78.7)	28 (80.0)	136 (89.5)	
Smoking status, n (%)				0.227
No	140 (74.9)	29 (82.9)	111 (73)	
Yes	47 (25.1)	6 (17.1)	41 (27)	
Hypertension, n (%)				0.673
No	139 (74.3)	27 (77.1)	112 (73.7)	
Yes	48 (25.7)	8 (22.9)	40 (26.3)	
Diabetes mellitus, n (%)				0.741
No	172 (92)	33 (94.3)	139 (91.4)	
Yes	15 (8.0)	2 (5.7)	13 (8.6)	
COPD, n (%)				1.000
No	179 (96.2)	33 (97.1)	146 (96.1)	
Yes	7 (3.8)	1 (2.9)	6 (3.9)	
Pulmonary tuberculosis, n (%)				0.691
No	177 (94.7)	34 (97.1)	143 (94.1)	
Yes	10 (5.3)	1 (2.9)	9 (5.9)	
BMI, kg/m^2^	22.43 (20.07, 24.34)	22.64 (21.09, 25.91)	22.27 (19.99, 24.15)	0.259
SBP, mmHg	129.00 (119.00, 138.00)	127.00 (115.00, 139.00)	130.00 (119.00, 138.00)	0.954
DBP, mmHg	78.71 ± 11.00	76.97 ± 10.82	79.11 ± 11.03	0.300
Hemoglobin, g/L	125.59 ± 15.36	121.60 ± 17.12	126.51 ± 14.84	0.088
Albumin, g/L	36.23 ± 4.56	36.32 ± 4.26	36.21 ± 4.63	0.898
WBC, ×10^9^/L	6.06 (4.99, 7.78)	4.65 (3.88, 5.65)	6.37 (5.22, 8.20)	<0.001
Neutrophil count, ×10^9^/L	4.18 (2.95, 5.38)	2.63 (2.07, 3.20)	4.62 (3.61, 5.87)	<0.001
Lymphocyte count, ×10^9^/L	1.33 (0.97, 1.76)	1.42 (1.28, 1.84)	1.26 (0.94, 1.73)	0.009
Monocyte count, ×10^9^/L	0.42 (0.31, 0.54)	0.35 (0.28, 0.45)	0.43 (0.33, 0.56)	0.009
Platelet count, ×10^9^/L	225.00 (169.00, 272.00)	176.00 (146.00, 206.00)	235.50 (184.00, 286.25)	<0.001
ALT, U/L	16.00 (11.00, 25.00)	15.00 (11.00, 26.00)	16.00 (11.00, 22.75)	0.759
AST, U/L	21.00 (17.00, 27.00)	20.00 (16.00, 27.00)	21.00 (17.00, 27.00)	0.605
TBil, μmol/L	10.30 (8.40, 14.00)	10.00 (7.60, 12.80)	10.30 (8.40, 14.15)	0.344
BUN, mmol/L	5.40 (4.30, 6.60)	5.70 (4.70, 7.10)	5.30 (4.23, 6.50)	0.151
Cr, μmol/L	64.30 (53.20, 71.90)	62.10 (49.10, 70.00)	64.65 (53.28, 72.33)	0.349
UA, μmol/L	294.00 (234.00, 351.30)	307.20 (242.90, 355.90)	290.70 (233.78, 350.33)	0.486
FBG, mmol/L	5.10 (4.76, 5.73)	5.09 (4.60, 5.44)	5.11 (4.78, 5.93)	0.115
TG, mmol/L	1.13 (0.83, 1.56)	1.21 (0.97, 1.90)	1.10 (0.81, 1.53)	0.102
TC, mmol/L	4.41 (3.81, 5.11)	4.32 (4.11, 5.11)	4.42 (3.77, 5.11)	0.852
HDL-C, mmol/L	1.18 (1.00, 1.43)	1.15 (1.01, 1.45)	1.20 (1.00, 1.425)	0.546
LDL-C, mmol/L	2.36 (1.91, 2.76)	2.17 (1.83, 2.80)	2.38 (1.92, 2.75)	0.919
Serum potassium, mmol/L	4.04 (3.74, 4.23)	4.00 (3.74, 4.17)	4.05 (3.75, 4.25)	0.419
Serum sodium, mmol/L	140.50 (139.10, 142.20)	140.20 (139.3, 142.2)	140.55 (139.00, 142.28)	0.864
Serum calcium, mmol/L	2.28 (2.19, 2.36)	2.26 (2.16, 2.35)	2.28 (2.20, 2.38)	0.174
ACM, n (%)				0.001
No	46 (24.6)	16 (45.7)	30 (19.7)	
Yes	141 (75.4)	19 (54.3)	122 (80.3)	

*SII*, systemic immune-inflammation index; *PR*, partial response; *SD*, stable disease; *PD*, progressive disease; *COPD*, chronic obstructive pulmonary disease; *BMI*, body mass index; *SBP*, systolic blood pressure; *DBP*, diastolic blood pressure; *WBC*, white blood cell count; *ALT*, alanine aminotransferase; *AST*, aspartate aminotransferase; *TBil*, total bilirubin; *BUN*, blood urea nitrogen; *Cr*, creatinine; *UA*, uric acid; *FBG*, fasting blood glucose; *TG*, triglycerides; *TC*, total cholesterol; *HDL-C*, high-density lipoprotein cholesterol; *LDL-C*, low-density lipoprotein cholesterol.

**Figure 1:**
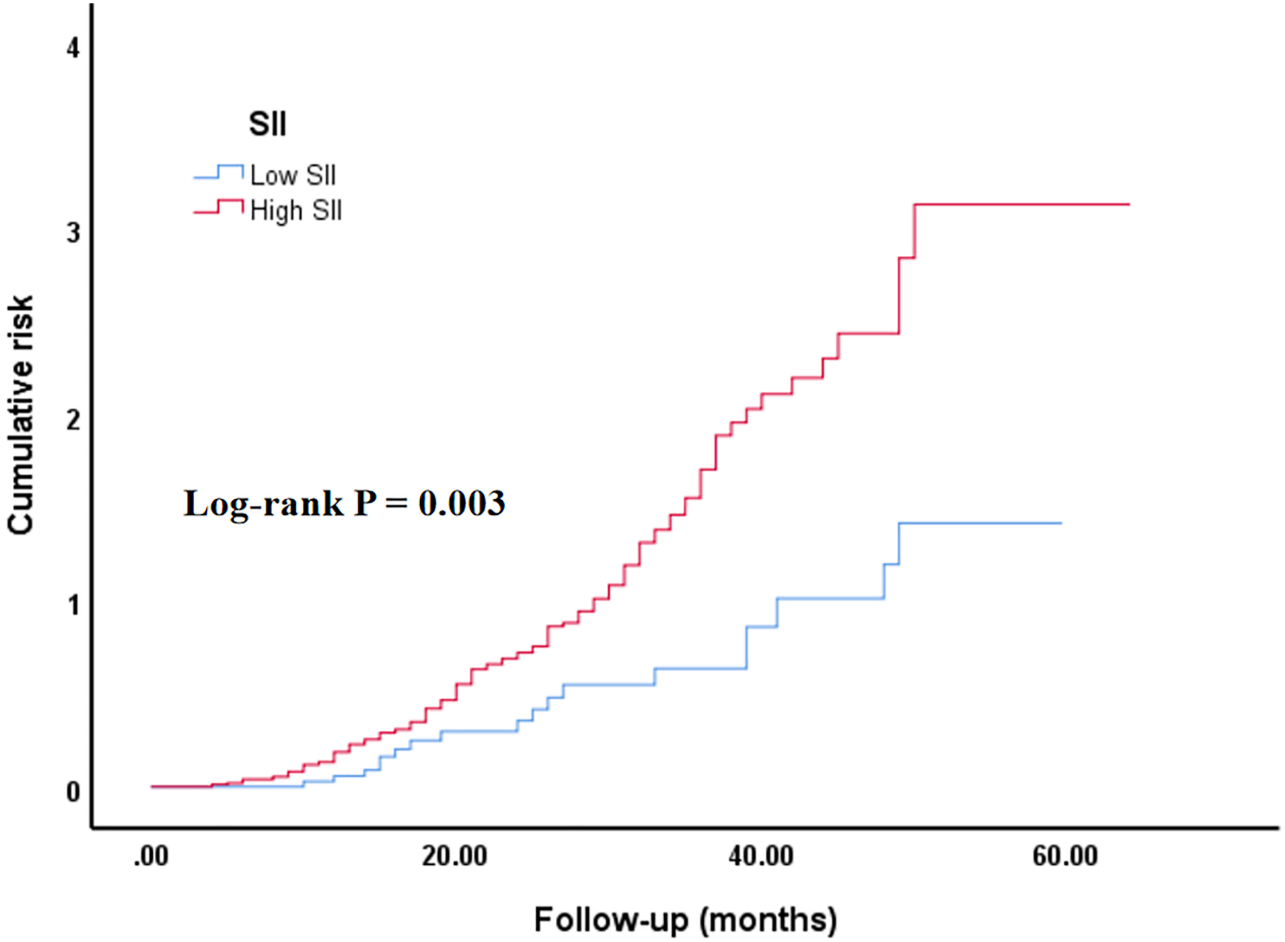
Kaplan–Meier curve of the cumulative risk of ACM stratified by SII level. Low SII group: SII ≤ 411.29; high SII group (SII > 411.29). SII, systemic immune-inflammation index. The cumulative risk represents the estimated probability of ACM over time, derived from the KM survival analysis.

Additionally, the cumulative ACM risk was significantly higher in the high SII group in comparison with the low SII group. KM survival curves demonstrated a persistent notable difference between the two groups from the early stages of follow-up (log-rank *p* = 0.003) (**Figure 1**).

### 3.2 Baseline characteristics according to ACM

As illustrated in **Table 2**, compared with the non-ACM group, the ACM group had notably reduced levels of LDL-C (*p* < 0.001), BUN (*p* = 0.035), TC (*p* = 0.044), albumin (*p* = 0.019), SC (*p* = 0.045), and elevated levels of disease progression (*p* < 0.001), WBC (*p* = 0.028), neutrophil count (*p* = 0.008), and monocyte count (*p* = 0.046). Additionally, no substantial differences between the two groups were found in clinical stage, site of metastasis, treatment modality, family history, treatment response, age, smoking status, pathological type, HP, DM, COPD, PTB, BMI, SBP, DBP, hemoglobin, lymphocyte count, platelet count, HDL-C, Cr, ALT, FBG, AST, TBil, UA, TG, SP, SS, and SII (*p* > 0.05).

**Table 2:** Baseline characteristics stratified by ACM.

Variable	No ACM	ACM	P-value
Age	62.00 (54.50, 73.00)	65.00 (55.00, 72.00)	0.640
Sex, n (%)			0.372
Male	20 (43.5)	72 (51.1)	
Female	26 (56.5)	69 (48.9)	
Family history, n (%)			1.000
No	45 (97.8)	138 (97.9)	
Yes	1 (2.2)	3 (2.1)	
Pathological type, n (%)			0.493
Adenocarcinoma	41 (89.1)	120 (85.1)	
Special subtype of adenocarcinoma	5 (10.9)	21 (14.9)	
Clinical stage, n (%)			0.531
Stage III	5 (10.9)	10 (7.1)	
Stage IV	41 (89.1)	131 (92.9)	
Site of metastasis, n (%)			0.278
Intrapulmonary metastasis only	29 (63.0)	76 (53.9)	
Extrapulmonary metastasis included	17 (37.0)	65 (46.1)	
Treatment modality, n (%)			0.751
Targeted therapy alone	27 (58.7)	79 (56.0)	
Combination therapy	19 (41.3)	62 (44.0)	
Treatment response, n (%)			0.366
PR	44 (95.7)	128 (90.8)	
SD+PD	2 (4.3)	13 (9.2)	
Disease progression, n (%)			<0.001
No	23 (50.0)	0 (0.0)	
Yes	23 (50.0)	141 (100.0)	
Smoking, n (%)			0.541
No	36 (78.3)	104 (73.8)	
Yes	10 (21.7)	37 (26.2)	
Hypertension, n (%)			0.482
No	36 (78.3)	103 (73.0)	
Yes	10 (21.7)	38 (27.0)	
Diabetes mellitus, n (%)			0.366
No	44 (95.7)	128 (90.8)	
Yes	2 (4.3)	13 (9.2)	
COPD, n (%)			1.000
No	45 (97.8)	134 (95.7)	
Yes	1 (2.2)	6 (4.3)	
Pulmonary tuberculosis, n (%)			0.710
No	43 (93.5)	134 (95.0)	
Yes	3 (6.5)	7 (5.0)	
BMI, kg/m^2^	23.13 (21.10, 25.41)	22.23 (19.81, 24.12)	0.063
SBP, mmHg	129.50 (114.50, 140.00)	129.00 (119.50, 137.50)	0.947
DBP, mmHg	78.39 ± 11.36	78.82 ± 10.91	0.821
Hemoglobin, g/L	126.91 ± 17.38	125.16 ± 14.68	0.502
Albumin, g/L	37.59 ± 4.37	35.79 ± 4.54	0.019
WBC, ×10^9^/L	5.22 (4.34, 7.09)	6.17 (5.14, 7.84)	0.028
Neutrophil count, ×10^9^/L	3.27 (2.61, 4.81)	4.32 (3.23, 5.54)	0.008
Lymphocyte count, ×10^9^/L	1.33 (1.08, 1.78)	1.33 (0.96, 1.77)	0.525
Monocyte count, ×10^9^/L	0.38 (0.27, 0.48)	0.43 (0.33, 0.55)	0.046
Platelet count, ×10^9^/L	218.50 (173.75, 292.50)	227.00 (167.00, 271.00)	0.799
ALT, U/L	14.00 (10.75, 23.50)	16.00 (11.00, 25.00)	0.270
AST, U/L	20.00 (16.00, 24.25)	21.00 (17.00, 27.00)	0.191
TBil, μmol/L	10.40 (8.48, 14.00)	10.20 (8.20, 13.95)	0.996
BUN, mmol/L	5.80 (4.70, 7.18)	5.10 (4.20, 6.55)	0.035
Cr, μmol/L	65.50 (53.68, 73.13)	63.40 (53.20, 71.55)	0.893
UA, μmol/L	316.05 (252.05, 368.75)	287.7 (226.35, 348.60)	0.076
FBG, mmol/L	5.23 (4.89, 5.64)	5.04 (4.72, 5.75)	0.395
TG, mmol/L	1.18 (0.81, 1.89)	1.11 (0.85, 1.52)	0.478
TC, mmol/L	4.62 (4.09, 5.33)	4.31 (3.72, 5.07)	0.044
HDL-C, mmol/L	1.15 (0.98, 1.32)	1.20 (1.02, 1.47)	0.092
LDL-C, mmol/L	2.63 (2.16, 3.31)	2.24 (1.83, 2.69)	<0.001
Serum potassium, mmol/L	4.00 (3.74, 4.09)	4.06 (3.75, 4.27)	0.135
Serum sodium, mmol/L	140.30 (139.18, 142.20)	140.50 (139.05, 142.35)	0.679
Serum calcium, mmol/L	2.33 (2.22, 2.40)	2.26 (2.18, 2.35)	0.045
SII	574.84 (380.67, 865.13)	692.71 (478.43, 1046.14)	0.045

*SII*, systemic immune-inflammation index; *PR*, partial response; *SD*, stable disease; *PD*, progressive disease; *COPD*, chronic obstructive pulmonary disease; *BMI*, body mass index; *SBP*, systolic blood pressure; *DBP*, diastolic blood pressure; *WBC*, white blood cell count; *ALT*, alanine aminotransferase; *AST*, aspartate aminotransferase; *TBil*, total bilirubin; *BUN*, blood urea nitrogen; *Cr*, creatinine; *UA*, uric acid; *FBG*, fasting blood glucose; *TG*, triglycerides; *TC*, total cholesterol; *HDL-C*, high-density lipoprotein cholesterol; *LDL-C*, low-density lipoprotein cholesterol.

### 3.3 Univariate Cox analysis of ACM

As illustrated in **Table 3**, univariate Cox analysis demonstrated that DM, the special types of ADC, WBC, neutrophil count, monocyte count, FBG, and SC were notably linked to ACM (*p* < 0.05). In contrast, other variables were not significantly related to ACM (≥ 0.05).

**Table 3:** Univariate Cox regression analysis of ACM.

Variable	HR	95% CI	*P*-值
Age	1.009	0.996 - 1.023	0.174
Gender			
Male	1.239	0.888 - 1.728	0.208
Female	Ref		
Family history	1.901	0.599 - 6.033	0.275
Pathological type			
Adenocarcinoma	Ref		
Special types of adenocarcinoma	2.180	1.360 - 3.495	0.001
Clinical stage			
Stage III	Ref		
Stage IV	1.640	0.861 - 3.125	0.132
Site of metastasis			
Intrapulmonary metastasis only	Ref		
Extrapulmonary metastasis included	1.229	0.879 - 1.716	0.228
Treatment modality			
Targeted therapy alone	Ref		
Combination therapy	1.098	0.785 - 1.536	0.586
Treatment response			
PR	0.689	0.388 - 1.222	0.203
SD+PD	Ref		
Disease progression	24.140	2.306 - 252.740	0.008
Smoking status	1.071	0.732 - 1.566	0.724
Hypertension	0.896	0.617 - 1.302	0.566
Diabetes mellitus	2.804	1.560 - 5.039	0.001
COPD	0.542	0.219 - 1.343	0.186
Pulmonary tuberculosis	0.952	0.444 - 2.038	0.898
BMI	0.978	0.930 - 1.028	0.379
SBP	1.005	0.994 - 1.016	0.364
DBP	1.004	0.989 - 1.020	0.606
Hemoglobin	0.993	0.982 - 1.004	0.194
Albumin	0.971	0.935 - 1.008	0.126
WBC	1.080	1.006 - 1.160	0.034
Neutrophil count	1.119	1.036 - 1.210	0.004
Lymphocyte count	0.738	0.532 - 1.023	0.068
Monocyte count	2.275	1.007 - 5.137	0.048
Platelet count	1.000	0.998 - 1.002	0.915
ALT	1.005	0.997 - 1.012	0.199
AST	1.008	0.992 - 1.025	0.311
TBil	0.993	0.961 - 1.025	0.648
BUN	0.241	0.855 - 1.040	0.943
Cr	1.007	0.998 - 1.016	0.125
UA	1.000	0.998 - 1.002	0.942
FBG	1.179	1.003 - 1.386	0.046
TG	1.010	0.791 - 1.291	0.934
TC	0.871	0.731 - 1.037	0.121
HDL-C	1.121	0.663 - 1.896	0.669
LDL-C	0.792	0.608 - 1.031	0.083
Serum potassium	1.246	0.796 - 1.951	0.336
Serum sodium	1.055	0.990 - 1.123	0.099
Serum calcium	1.032	1.008 - 1.057	0.008

*SII*, systemic immune-inflammation index; *PR*, partial response; *SD*, stable disease; *PD*, progressive disease; *COPD*, chronic obstructive pulmonary disease; *BMI*, body mass index; *SBP*, systolic blood pressure; *DBP*, diastolic blood pressure; *WBC*, white blood cell count; *ALT*, alanine aminotransferase; *AST*, aspartate aminotransferase; *TBil*, total bilirubin; *BUN*, blood urea nitrogen; *Cr*, creatinine; *UA*, uric acid; *FBG*, fasting blood glucose; *TG*, triglycerides; *TC*, total cholesterol; *HDL-C*, high-density lipoprotein cholesterol; *LDL-C*, low-density lipoprotein cholesterol.

### 3.4 Multivariate Cox regression analysis of SII and ACM

As illustrated in **Table 4**, SII, analyzed both as a categorical and continuous variable, was notably associated with ACM in the unadjusted model 1. Compared with the low SII group, a 2.027-fold increase in ACM risk was found in the high SII group (HR = 2.027; 95% CI = 1.242–3.307; *p* = 0.005). In model 2, which controlled for DM and pathological type, a 1.999-fold elevated ACM risk was observed in the high SII group (HR = 1.999; 95% CI = 1.225–3.262; *p* = 0.006) in contrast to the low SII group. Furthermore, a higher SII remained correlated with an elevated ACM risk in model 3, which controlled for DM, pathological type, FBG, WBC, neutrophil count, and SC. In this model, in contrast to the low SII group, a 1.985-fold greater ACM risk was found in the high SII group (HR = 1.985; 95% CI = 1.216–3.240; *p* = 0.006). These findings suggested that SII may act as an independent biomarker for assessing unfavorable prognosis.

**Table 4:** Multivariate Cox regression analysis of SII and ACM.

Variable	Model 1^a^			Model 2^b^			Model 3^c^		
	HR	95% CI	*P*-value	HR	95% CI	*P*-value	HR	95% CI	*P*-value
SII	1.000	1.000–1.000	0.001	1.000	1.000–1.000	0.014	1.000	1.000–1.000	0.036
Log_10_SII	2.339	1.306–4.192	0.004	2.000	1.108–3.608	0.021	1.883	1.039–3.411	0.037
Low SII	Ref			Ref			Ref		
High SII	2.027	1.242–3.307	0.005	1.999	1.225–3.262	0.006	1.985	1.216–3.240	0.006

*SII*, systemic immune-inflammation index.

a Unadjusted.

b Adjusted for diabetes and pathological type.

c Adjusted for diabetes, pathological type, fasting glucose, white blood cell count, monocyte count, and calcium.

### 3.5 Subgroup analysis

As illustrated in **Table 5**, multivariate Cox analysis suggested that an elevated SII was associated with a higher ACM risk among both women and men. The association was more pronounced in the male subgroup (HR = 3.245; 95% CI = 1.416–7.435; *p* = 0.005). Compared with the low SII group, the ACM risk in the high SII group was 2.036 times higher in the female subgroup (HR = 2.036; 95% CI = 1.006–4.121; *p* = 0.048). In the age subgroup, a high SII was notably associated with an elevated ACM risk among older patients aged above 65 (HR = 2.675; 95% CI = 1.372–5.146; *p* = 0.004), indicating that SII may be a stronger indicator for predicting mortality risk in older populations.

**Table 5:** Subgroup analysis of SII and ACM.

Variable	High SII vs. low SII		
	HR	95% CI	*P*-value
Gender			
Male	3.245	1.416–7.435	0.005
Female	2.036	1.006–4.121	0.048
Age			
< 65 years	1.708	0.747–3.907	0.205
≥ 65 years	2.657	1.372–5.146	0.004

The subgroup analysis was adjusted for diabetes, pathological type, fasting glucose, white blood cell count, monocyte count, and calcium. *SII*, systemic immune-inflammation index.

### 3.6 Sensitivity analysis

As illustrated in **Table 6**, after excluding individuals with special types of ADC, a higher SII was still associated with an elevated ACM risk. Compared with the low SII group, a 1.989-fold increase in ACM risk was observed in the high SII group (HR = 1.989; 95% CI = 1.182–3.347; *p* = 0.010). After excluding stage III LC patients, SII—whether analyzed as a categorical or continuous variable—remained notably associated with ACM, with the high SII group having a 1.824-fold increased ACM risk (95% CI = 1.103–3.015; *p* = 0.019) compared with the low SII group. After excluding diabetic patients, a higher SII was notably linked to an elevated ACM risk, with the high SII group having a 2.106-fold greater ACM risk (95% CI = 1.255–3.533; *p* = 0.005) compared with the low SII group. These sensitivity analyses indicated that the association between SII and ACM remained significant after excluding individuals with specific types of ADC, stage III disease, or DM. These results confirm the robustness of SII as an independent indicator for predicting ACM.

**Table 6:** Multivariate Cox regression analysis of SII and ACM: sensitivity analysis.

Variable	HR	95% CI	*P*-value
Exclusion of a special type of adenocarcinoma			
SII	1.000	1.000–1.001	0.045
Log_10_SII	2.179	0.935–5.077	0.071
Low SII	Ref		
High SII	1.989	1.182–3.347	0.010
Exclusion of stage III patients			
SII	1.000	1.000–1.000	0.023
Log_10_SII	1.875	1.005–3.497	0.048
Low SII	Ref		
High SII	1.824	1.103–3.015	0.019
Exclusion of diabetic patients			
SII	1.000	1.000–1.001	0.002
Log_10_SII	2.450	1.292–4.648	0.006
Low SII	Ref		
High SII	2.106	1.255–3.533	0.005

The multivariate regression analysis was adjusted for diabetes, pathological type, fasting glucose, white blood cell count, monocyte count, and calcium. *SII*, systemic immune-inflammation index.

### 3.7 Prognostic value of SII for all-cause mortality

**Figure 2:**
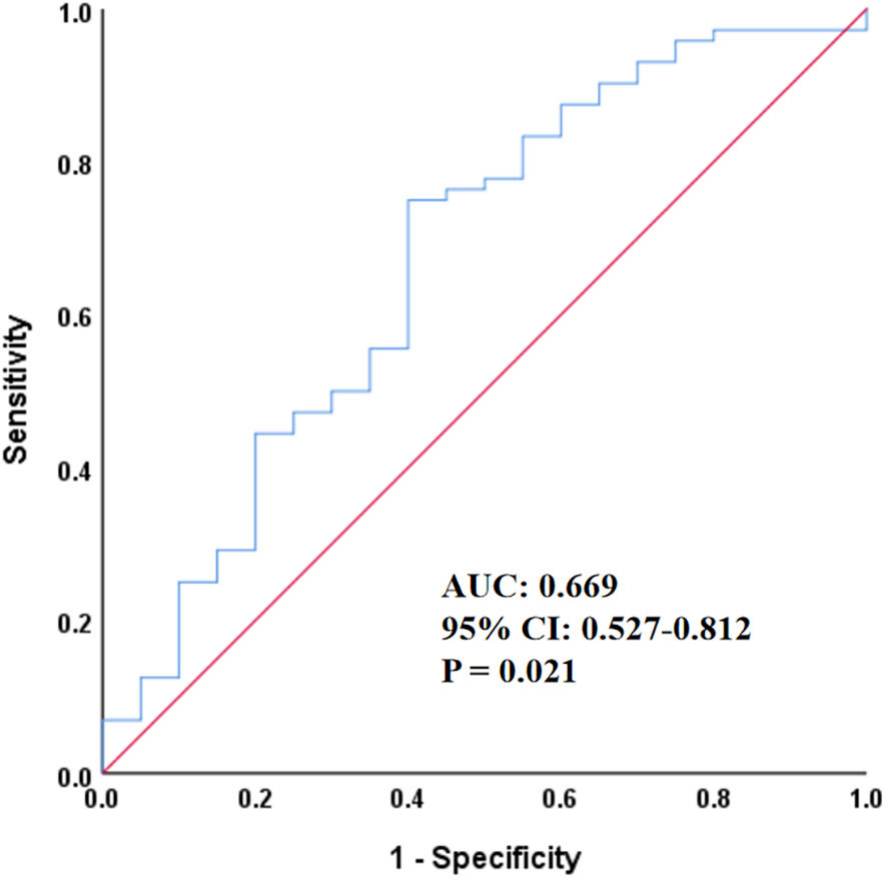
Receiver operating characteristic curve of SII for predicting all-cause mortality. SII, systemic immune-inflammation index; AUC, area under the curve; CI, confidence interval.

As shown in **Figure 2**, ROC curve analysis was performed to evaluate the ability of SII to predict ACM in patients with stages IIIB–IV EGFR-mutated lung adenocarcinoma. The area under the curve (AUC) was 0.669 (95% CI = 0.527–0.812; *p* = 0.021), indicating a modest but statistically significant predictive value.

## 4 Discussion

Lung cancer ranks first in both incidence and mortality worldwide, and adenocarcinoma is its most common histological subtype, characterized by complex biological features. The early symptoms of lung adenocarcinoma are typically unnoticeable, so a majority of individuals are diagnosed only after clinical signs appear, usually at an advanced stage. As a result, these patients often have poor overall survival (OS) outcomes. Therefore, exploring the factors influencing lung adenocarcinoma prognosis holds great clinical significance.

Increasing evidence has revealed that inflammation plays a crucial role in the tumor biology of EGFR-mutated NSCLC. Chronic inflammatory signaling can promote tumor initiation, clonal evolution, and therapeutic resistance through multiple mechanisms. EGFR activation has been shown to interact with inflammatory pathways such as NF-κB and IL-6/STAT3, leading to enhanced tumor proliferation, angiogenesis, and an immunosuppressive microenvironment (9, 10). These interactions create a feed-forward loop in which EGFR signaling induces the release of proinflammatory cytokines, while inflammation, in turn, sustains EGFR pathway activation and tumor progression.

SII, which consists of lymphocyte, platelet, and neutrophil counts, can comprehensively and objectively assess the dynamic balance between immune status and inflammatory response (11). Hu et al. (12) first proposed the concept of SII and found that a higher SII in individuals with liver cancer was notably associated with an elevated risk of recurrence. Existing evidence has demonstrated that SII is linked to the prognosis of multiple solid tumors, including pancreatic cancer, esophageal cancer, and gastric cancer (13–15). Moreover, systemic inflammatory indices such as the SII reflect the dynamic balance between host immunity and tumor-related inflammation. Elevated SII indicates increased neutrophil- and platelet-driven inflammatory activity, along with suppressed lymphocyte-mediated immune surveillance, which may further enhance the aggressiveness of EGFR-mutant tumors (8). Recent studies suggest that chronic inflammation contributes to epithelial–mesenchymal transition (EMT), resistance to EGFR tyrosine kinase inhibitors (EGFR-TKIs), and metastatic potential in EGFR-mutant NSCLC (16). Berardi et al. (17) found that individuals with advanced NSCLC and high pretreatment SII had significantly shorter OS following first-line chemotherapy or targeted therapy. Their finding suggests that SII can independently predict unfavorable outcomes. Our results align with those of Berardi et al. (17), indicating that SII is notably related to ACM, whether treated as a continuous or categorical variable. Biswas et al. (18) have demonstrated that SII is a critical marker for predicting OS and progression-free survival (PFS) among stage III locally advanced NSCLC patients. Other researchers (19) suggested that individuals with stage IIIA/N2 NSCLC and elevated SII before treatment may require more intensive adjuvant therapies. Moreover, a recent study (20) identified SII as the strongest predictor of pulmonary complications following LC resection. Tong et al. (8) demonstrated that high SII is associated with chemoresistance among individuals with advanced NSCLC undergoing first-line platinum-based chemotherapy. Patients with SII ≥ 660 tend to have lower response rates to chemotherapy and more advanced clinical stages than those with SII< 660. These findings indicate that chemoresistance remains a major challenge in cancer treatment. SII ≥ 660 can predict poor prognosis in advanced NSCLC, and pretreatment SII is an independent indicator for predicting OS, outperforming other peripheral blood biomarkers. Therefore, pretreatment SII may help predict patients’ responsiveness to chemotherapy. A retrospective study (7) examined the performance of the blood-based SII in 203 EGFR-mutant advanced lung ADC patients who received first-line EGFR-TKIs. Their results indicated that individuals with SII ≥ 1,066.935 have a greater likelihood of poor Eastern Cooperative Oncology Group performance status (ECOG-PS). In their multivariate analysis, SII was an independent biomarker for assessing OS (HR = 2.802; 95% CI = 1.659–4.733; *p* < 0.001) and PFS (HR = 2.577; 95% CI = 1.677–3.958; *p* < 0.001). Their findings suggest that SII is notably related to prognosis in EGFR-mutant lung ADC patients receiving first-line EGFR-TKIs, with higher SII indicating greater tumor burden, more profound immunosuppression, and poorer outcomes.

The specific mechanisms by which elevated SII leads to lower survival in cancer patients remain unestablished. It is generally believed that higher SII reflects a decline in lymphocyte count and an increase in platelet and neutrophil counts. Neutrophils induce DNA damage, promote angiogenesis, and activate endothelial and stromal cells by secreting a series of proangiogenic factors and proinflammatory molecules. These processes can enhance tumor cell invasion and metastatic potential, ultimately leading to distant metastasis (21). Platelets may impact tumor progression and metastasis by secreting platelet-derived growth factor, transforming growth factor beta, and vascular endothelial growth factor (22). As major effector cells of the immune system, lymphocytes are essential for suppressing tumor cell invasion, proliferation, and migration, and are critical for tumor immune surveillance (23). Impaired lymphocyte function or reduced lymphocyte counts can compromise immune surveillance, thereby weakening tumor control and ultimately resulting in a worse prognosis. Therefore, a high SII reveals an enhanced inflammatory response and a weakened immune defense, suggesting a poorer clinical outcome.

However, some limitations should be acknowledged. Firstly, selection bias may be present, and the generalizability of the findings requires further validation, as this is a single-center retrospective study with a small sample size. Secondly, because the data were derived from real-world clinical records, some potential confounders—including nutritional status and unmeasured infections affecting inflammatory markers—are not fully accounted for. Due to incomplete information on resistance mechanisms for some patients, treatment-related variables—including the line of EGFR-TKI therapy (first, second, or third line), mutation subtype (e.g., exon 19 deletion, L858R, T790M), and the presence of acquired resistance mutations upon disease progression—were not analyzed in the subgroup models. Thirdly, as SII was assessed only at baseline, longitudinal changes and dynamic monitoring were not available, which may limit temporal interpretation. External validation using independent cohorts is required to confirm generalizability. Future prospective multicenter studies integrating serial SII measurements and molecular biomarkers are warranted. Additionally, since this study was retrospectively designed, ACM was defined as death events that occurred by the end of follow-up (1 January 2025). It was not feasible to restrict ACM to specific time intervals (e.g., 2-, 3-, or 5-year postdiagnosis) because many patients had been discharged for more than 5 years when data collection began. This temporal limitation arises inherently from the retrospective nature of the study. Future prospective cohort studies with planned follow-up at fixed time points after diagnosis (such as 2, 3, and 5 years) are warranted to provide more granular prognostic evidence and to minimize recall bias.

Future larger prospective studies conducted across multiple centers are required to further explore its prognostic value. Although subject to certain limitations, this study revealed the value of SII in independently predicting stages IIIB–IV EGFR-mutated lung adenocarcinoma. In clinical practice, weighing the cost–benefit ratio is a critical factor in formulating cancer treatment plans. Notably, SII, derived from routine laboratory parameters, is a readily accessible and cost-effective marker that may assist in guiding clinical decision-making. It holds potential as a valuable indicator for assessing prognosis in clinical practice.

## 5 Conclusion

Our findings suggest that SII is associated with all-cause mortality in patients with stages IIIB–IV EGFR-mutated lung adenocarcinoma, regardless of mutation subtype, indicating the potential of SII as a prognostic blood biomarker. It could provide valuable references for risk assessment and individualized treatment planning. Larger prospective studies conducted across multiple centers are required to verify the value of SII and to develop more accurate prognostic models incorporating dynamic monitoring and multiomics indicators, thereby enhancing risk stratification and guiding clinical decision-making in LC management.

## Data Availability

The original contributions presented in the study are included in the article/supplementary material. Further inquiries can be directed to the corresponding author.
